# Review of Plastic Surgery Biomaterials and Current Progress in Their 3D Manufacturing Technology

**DOI:** 10.3390/ma13184108

**Published:** 2020-09-16

**Authors:** Wei Peng, Zhiyu Peng, Pei Tang, Huan Sun, Haoyuan Lei, Zhengyong Li, Didi Hui, Colin Du, Changchun Zhou, Yongwei Wang

**Affiliations:** 1 Department of Palliative Care, West China School of Public Health and West China Fourth Hospital, Sichuan University, Chengdu 610041, China; hxnyyyg@163.com; 2 Occupational Health Emergency Key Laboratory of West China Fourth Hospital, Sichuan University, Chengdu 610041, China; 3 Department of Thoracic Surgery, West China School of Medicine, West China Hospital, Sichuan University, Chengdu 610041, China; pengzy94@sina.com; 4 Department of Burn and Plastic Surgery, West China School of Medicine, West China Hospital, Sichuan University, Chengdu 610041, China; tangpp94@sina.cn (P.T.); lizydd@sina.com (Z.L.); 5 National Engineering Research Center for Biomaterials, Sichuan University, Chengdu 610064, China; Sunhuan_1995@163.com (H.S.); leihaoyuan@stu.scu.edu.cn (H.L.); changchunzhou@scu.edu.cn (C.Z.); 6 Innovatus Oral Cosmetic & Surgical Institute, Norman, OK 73069, USA; ddhui423@gmail.com (D.H.); 99dumeng@gmail.com (C.D.)

**Keywords:** biomaterial, plastic surgery, injectable material, prosthesis material, 3D manufacturing

## Abstract

Plastic surgery is a broad field, including maxillofacial surgery, skin flaps and grafts, liposuction and body contouring, breast surgery, and facial cosmetic procedures. Due to the requirements of plastic surgery for the biological safety of materials, biomaterials are widely used because of its superior biocompatibility and biodegradability. Currently, there are many kinds of biomaterials clinically used in plastic surgery and their applications are diverse. Moreover, with the rise of three-dimensional printing technology in recent years, the macroscopically more precise and personalized bio-scaffolding materials with microporous structure have made good progress, which is thought to bring new development to biomaterials. Therefore, in this paper, we reviewed the plastic surgery biomaterials and current progress in their 3D manufacturing technology.

## 1. Introduction

The term “plastic surgery” was first coined by German surgeon Karl Ferdinand von Greffy (1787–1840) in 1818 [1]. At the end of the 19th century, the rise of feminism after the first industrial revolution accelerated the development of cosmetic surgery [2]. In the early 20th century, the two world wars caused a lot of tissue defects and deformities, prompting some doctors, mainly oral and maxillofacial surgeons and otolaryngologists, to conduct research on plastic surgery [3,4]. Therefore, plastic surgery has made unprecedented progress.

At present, the theories and methods of plastic and aesthetic medicine have received a lot of innovations and applications, with the related plastic cosmetic biomaterials and the application of new technologies developing rapidly. With the improvement of people’s living standards and the pursuit of “beauty” more and more, the safety of plastic cosmetic materials has gradually been paid attention to [5]. The scope of clinical application of local minimally invasive plastic surgery and non-surgical methods is getting wider and wider [6,7,8]. Biomaterials are native or synthetic polymers that act as carriers for drug delivery or scaffolds for tissue regeneration [9]. Biomaterials used in plastic and aesthetic surgery are called plastic cosmetic biomaterials, which can be classified into injectable biomaterials and prosthesis materials by material use. Nowadays, safer and safer biomaterials and new technologies are widely employed in the plastic surgery industry. As a biomaterial used in the plastic surgery industry, it is necessary to meet the following conditions [10,11,12]: (1) Good tissue compatibility; (2) no allergic reaction, non-pyrogenic source; (3) not carcinogenic or teratogenic; (4) non-microbial survival matrix; (5) having a certain combination ability with the host organization; (6) not causing inflammation or foreign body reaction; (7) non-antigenic, not causing immune or tissue-related diseases; (8) easy to disinfect and store; (9) having proper liquidity; (10) easy to shape and fix after being placed into the host, with the effect being long-lasting or permanent.

Recently, three-dimensional (3D) bioprinting, based on tissue engineering and stem cell research, using living cells and other active components as printing materials have made huge progress and can further realize the formation of bioactive tissues or organs in vitro [13,14]. The three-dimensional manufacturing technology has brought a new prospect for the application of biomaterials in plastic surgery. Therefore, this paper reviews the latest advances in the research on biomaterials related to plastic surgery and current progress in their 3D manufacturing technology.

## 2. Classification of Plastic Cosmetic Materials

### 2.1. Plastic and Cosmetic Materials Classified by Source

**Figure 1:**
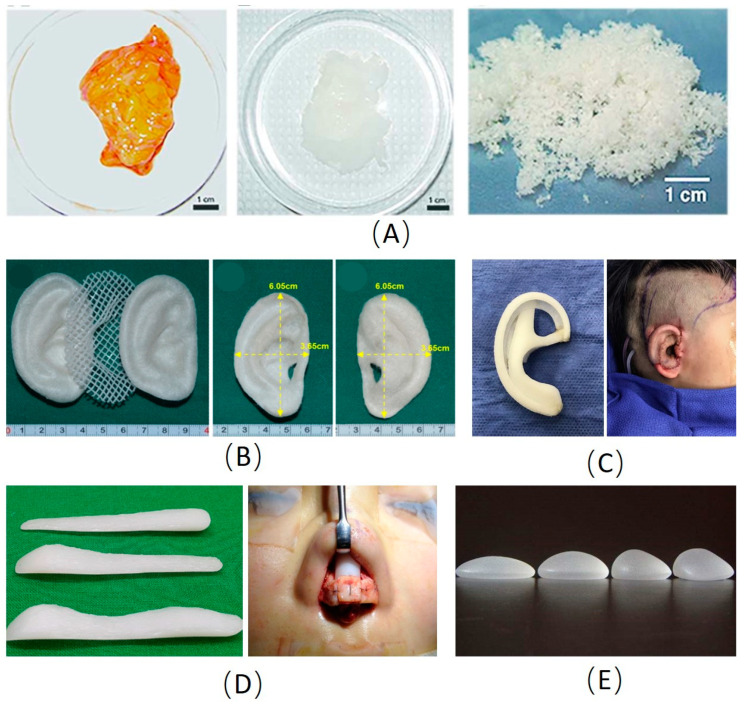
Examples of main materials used in plastic and cosmetic surgery. (**A**) Images of native and decellularized adipose tissue [20]. (**B**) The pre-shaped PLA inner core sandwiched between the pair of pre-shaped PGA layers for ear reconstruction [58]. (**C**) The porous polyethylene scaffold for ear reconstruction [42]. (**D**) The silicone scaffold for rhinoplasty [38]. (**E**) The silicone prostheses for breast enhancement. PLA: Polylactic acid; PGA: Polyglycolic acid.

Plastic cosmetic materials can also be divided into natural biomaterials and synthetic materials according to the source of the materials, with synthetic materials including organic polymer materials and inorganic non-metallic materials (Table 1 and Table 2, and Figure 1).

#### 2.1.1. Natural Biomaterials

**Bio-protein glue.** Medical bio-protein glue, also known as fibrin adhesive, is mainly composed of fibrinogen, thrombin, calcium chloride, and other substances, having a similar effect to the final stage of the coagulation process [15]. It has good compatibility with human tissues and does not produce irritation and toxic side effects [15,16]. Patients can reabsorb bio-protein glue after a few days of use to promote the growth and repair of their tissues. In burn surgery, small vein bleeding often occurs in wounds. The application of bio-protein glue can reduce the amount of bleeding, save the time of hemostasis, reduce the leakage of body fluids, shorten the operation time, and reduce the occurrence of complications such as infection [17,18].

**Decellularized tissue.** The acellular tissue matrix is a product of the decellularization process including a chemical or physical method for removing antigens associated with rejection in tissue transplantation [19,20]. It is a new type of biomaterial and is mainly used for the repair of cosmetic injury tissues, especially skin transplantation after burns. It will not produce a rejection reaction in the human body, and will gradually grow with the growth of the body, creating hope for the recovery of burn patients, which is irreplaceable by other artificial materials [12,19,21,22]. Extracellular matrix (ECM) scaffolds prepared from decellularized tissues have been used in a variety of clinical applications to promote constructive and functional tissue remodeling [23,24]. These ECM materials can be dissolved and subsequently manipulated to form a hydrogel, which is a culture medium comparable to collagen or matrix gel and can also be used as an injectable material to fill irregular shape defects [23]. The mechanism of ECM hydrogel in guiding cell behavior and affecting remodeling results is only partially understood, but likely include structural and biological signals retained from natural source tissues. Jackson et al. [25] used ECM with epidermal stem cells to treat patients with burns, chronic wounds, and achieved satisfactory results. Pati et al. [22] have developed a novel acellular ECM bioink for cell carrier bioprinting, which can provide an optimized microenvironment for tissue growth with a three-dimensional structure. Therefore, the ECM is a complex and dynamic microenvironment composed of a variety of extracellular molecules secreted by cells. However, due to the complexity of ECM regulating cells and a large number of participants, many tissue-specific pathways remain unclear [26]. More work needs to be done before the ECM can be widely used in clinical applications.

**Collagen** exists in the form of collagen fibers in the body. Collagen has the following advantages as a facial soft tissue filling material in plastic and cosmetic surgery: Good biocompatibility; can interact with cells; good mechanical properties; moisture retention; low immunogenicity; biodegradability [27]. After collagen is injected into the body, it is usually gradually absorbed by the body within 4–6 months, so collagen can also be used for surgical sutures [28].

**Hyaluronic acid (HA)** is a naturally occurring glycosaminoglycan, which is usually extracted from bacteria or cockscomb. HA has shown low immunogenicity in preclinical studies, and several HA materials have been approved for clinical use [29]. However, due to the quick degradation and requirement of repeated injections, HA is usually used as a temporary filler to induce tissue repair [30]. At present, there are two methods to degrade the degradation rate of HA, one is the double crosslinking method [30], the other is to combine HA with gelatin, chitosan, cellulose, and other biopolymers or synthetic polymers [31,32]. Compared with pure HA, the combination of HA and gelatin showed good biocompatibility and lower degradation rate [33]. Between January 2016 and June 2018, Segreto et al. [34] injected HA into 70 cosmetic patients and followed up on their clinical efficacy, adverse reactions, duration of efficacy, etc. The results show that HA rhinoplasty is safe, effective, and convenient, lasts 6–12 months, and eventually degrades into carbon dioxide and water. Although the allergic reaction induced by HA is weak, there are still local pain, bruises, and temporary edema [35]. HA injection rhinoplasty is a surgical method, which has the characteristics of effectiveness, safeness, wide indication, small trauma, and short recovery time, and thus it is a good auxiliary method for rhinoplasty [34,36].

#### 2.1.2. Organic Polymer

**Silicone.** Silicone was used clinically in the mid-20th century. It has the advantages of heat resistance, cold resistance, non-toxicity, biological aging resistance, physiological inertia, little response to human tissues, and good physical and mechanical properties, and therefore it meets the requirements for medical-grade polymer materials, and its application in the medical field is more and more extensive. Nowadays, medical silicone products have been applied to various parts of the human body, such as to correct nasal deformities or perform skull cosmetic surgery and repair internal organs. In addition to being implanted in the body, silicone can also be used as a prosthesis to repair the body surface. Silicone has the advantages of soft texture, moderate strength, strong operability, and excellent biocompatibility, and it has gradually become the first choice for the production of prostheses for facial defects [37,38]. It is used to treat maxillofacial defects and external ear defects caused by malignant tumors and trauma. These repairs improve the patient’s physiological functions such as chewing and pronunciation and alleviate some psychological disorders caused by maxillofacial injuries [39]. Moreover, silicone gels and hydrogels can be used as breast implants. However, the use of silicone as an implant material in the body still has deficiencies, such as strong hydrophobicity, poor antibacterial properties, and aging problems [40,41].

**Expanded Polytetrafluoroethylene (ePTFE).** The ePTFE material is an inert polymer tissue filling material with stable physical and chemical properties [42]. The ePTFE has an important application value in plastic surgery because ePTFE has the following characteristics.

- Good biological scaffold for cell growth. After implanting the ePTFE material, an histological observation showed that there was more tissue cells and giant cells on the surface of the material, the pores of the material were filled with a collagen matrix, the fibroblasts and functional capillaries attached to the surface of the material, and the tissue adheres to the surface of the material and grows in the pores of the material. Although the microporous structure of the material is firmly integrated into the tissue, it does not form a fibrous cystic structure and can be completely removed [43].
- Good biocompatibility. The ePTFE material has good biocompatibility, only a slight tissue reaction such as skin redness of the implant site [41], and good soft tissue stability [43,44].
- Good physical properties. The ePTFE material is soft, with similar elasticity and hardness as a soft tissue, and has good tensile strength [45].
- Good clinical application effect. Clinical follow-up observations showed that there were no long-term complications such as chronic inflammation, absorption, atrophy, and capsule formation. The hardness cannot only meet the support requirements of the local tissue structure but also have a certain degree of toughness, so as to obtain a natural and real feeling. Many pieces of the literature report the application of ePTFE in plastic surgery [43].

**Polymethyl methacrylate (PMMA)** exists in two forms: One is thermosetting, a hard pre-shaped graft; the other is cold-setting and can be shaped during surgery. The latter can release heat up to 70% during polymerization. Therefore, cold saline is used to rinse the graft continuously to avoid scalding of surrounding tissues. It is worth noting that this process may cause complications such as cardiac arrest and arrhythmia [46]. Typical foreign body reaction and fibrous tissue cyst are the main complications of the surrounding tissue implanted with methyl methacrylate [47]. The distance between the implant and the incision should be kept above 1 cm, with skin grafting and deep scars avoided to reduce the possibility of infection and exposure. Methyl methacrylate has X-ray penetration and good long-term tissue tolerance. In orthopedic surgery, methyl methacrylate containing gentamicin and muscle flaps are transplanted simultaneously to treat infectious long bone fractures [48]. Methyl methacrylate can also be used to augment the forehead and repair the chest wall [49,50]. In the early 1990s, polymethyl methacrylate (PMMA) particles were used as injectable filler materials, called Artecolll microspheres, which were made by mixing PMMA microspheres with 3.5% bovine collagen [51]. It is injected deep into the skin and is mainly used to treat facial wrinkles, fill in acne scars, correct nipple depressions, highlight chins, and swell cheeks. It contains collagen, so allergies should be tested before injection. Common complications of Artecolll injection include bead protrusions, allergic reactions, telangiectasia, and granuloma formation [51]. Since these fillers are permanent, they are difficult to remove after implantation, so the local injection of corticosteroids is the main treatment.

The trade name of high-density polyethylene is Medpor. It is white, with its surface being rough, convergent, and porous; it has a certain flexibility and relatively incompressibility, and can be formed by knife carving; it has a plate shape of 1.5–10 mm with different thicknesses, and can also be made according to different needs and prostheses of the jawbone and cheekbones; it has poor mechanical properties and is not suitable for load-bearing parts [52]. High-density polyethylene has good biocompatibility. After implanting it, fiber or bone tissue and blood vessels can grow into the hole, and the foreign body reaction is very light. There is a certain degree of bone conduction into the bone, which can repair the cheeks, orbital arch, orbital floor, upper and lower jaws, cheekbones, temporal, and ears [53,54,55]. Infection is the biggest problem it encounters in clinical application, so the disinfection of materials before surgery should be strict, and they should be placed in physiological saline containing antibiotics before use, so that the antibiotics after surgery can slowly dissolve in the pores, reducing the possibility of infection. Since these materials are easily contaminated with bacteria, patients must use antibiotics throughout the body before surgery [52,56].

Both polylactic acid (PLA) and polyglycolic acid (PGA) are aliphatic polyester biodegradable materials. Polylactic acid material is hydrolyzed in the body to generate CO_2_ and H_2_O [57]. Glycolic acid formed after the degradation of polyglycolic acid in the body can also participate in metabolism and excretion in the body [57]. Synthetic polylactic acid and polyglycolic acid can be made into surgical absorbable sutures, bone fixation plates, screws, etc. by drawing or by processing methods such as at a certain temperature, pressure, or with mold [58]. The copolymer of polylactic acid and polyglycolic acid is a suture called Vicryl. The tissue reaction is very small, and the tensile strength drops to 60% and 75% after being left for two weeks; after a month, the tension completely disappeared, and all absorbed after three months. This kind of suture is mainly used for dermal suture [59,60].

#### 2.1.3. Ceramic Materials

**Hydroxyapatite** has good biocompatibility. Being implanted in the body, it will not stimulate and repel the tissues, and the calcium ions and phosphate ions in the hydroxyapatite material will be freed to participate in the calcium and phosphorus cycle balance of human tissues. On the one hand, it is continuously absorbed by tissues; on the other hand, it also continuously produces new calcium and phosphorus salts, which have bone inducibility, bone guidance, and excellent mechanical strength [61]. The degree of hydroxyapatite absorption is strongly dependent on its crystallinity. It has a rather low rate of biodegradation, especially compared to other types of bioactive ceramics such as tricalcium phosphate (TCP). In plastic surgery, it is used as a substitute for large area bone defects such as oral and maxillofacial regions and substitutes for teeth in the oral cavity [62,63]. Although pure hydroxyapatite can become a substitute for human hard tissue, the simple hydroxyapatite material has the disadvantages of high brittleness, low strength, and poor toughness. Some composite materials formed by compounding other materials with hydroxyapatite can greatly improve the overall performance of hydroxyapatite. Fouad et al. [64] combined polyethylene scaffolds and hydroxyapatite, and implanted the composite material into the rat’s bone defect. The results show that the composite material has good biocompatibility and bone conductivity and that it maintains the plasticity of polyethylene.

**Bioglass materials** can form chemical bonds between the bone and bone tissue interface to induce bone repair and regeneration, which are mainly used for repair of the oral cavity, orthopedics, and plastic surgery. Singh et al. [65] reported the use of bioglass and the autogenous iliac crest in orthopedics by fabricating polyelectrolyte complexation mediated composite scaffold of chitosan and chondroitin sulfate incorporated with nano-sized bioglass. The results showed that the bioglass implanted scaffold facilitated tissue regeneration and integration with native bone tissue. Although bioglass has good biocompatibility and bone binding ability, it has low mechanical strength, high brittleness, poor bending strength, and compressive strength, which limits its application [66]. The combination of bioglass and metal materials not only changes the bioactive surface due to bioglass coating but also improves the mechanical strength due to the metallic core.

### 2.2. Classification According to Material Use

Plastic and cosmetic materials can be divided into prosthetic materials and injection materials according to their uses. Rhinoplasty prosthesis, chin prosthesis, temporal (temple) prosthesis, and breast augmentation prosthesis are all commonly used plastic prostheses. Injection materials are mainly used for tissue repair, deformity correction, and facial repair treatment. It is one of the trends in the development of plastic surgery clinical technology and is mainly derived from human, animal, and bacterial derivatives and compounds. There are several mechanisms of in situ gelations of injectable biomaterials by a physical method such as temperature, pH, electric field and magnetic field, and chemical method such as Schiff base crosslinked gelation, Michael addition crosslinked gelation, and enzyme-catalyzed crosslinked gelation [67,68]. Compared with the physical method, the chemical crosslinked gelation has the advantages of stable structure, high strength, good controllability, and easy operation. However, due to the involvement of other substances and chemical reactions, it will have a greater impact on cells [67,68,69]. The disadvantage of injection material is that it will cause unstable reactions and tissue allergic reactions after injection. At present, plastic and cosmetic injection materials are mainly used in plastic surgery and cosmetic surgery, specifically manifested as breast augmentation, rhinoplasty, face-lift, wrinkles, etc. Collagen, autologous fat, hyaluronic acid, botulinum toxin, Ivanol, etc. have been widely used in plastic surgery clinical operations.

#### 2.2.1. Prosthetic Materials

Prosthetic materials play an important role in plastic surgery. By filling prosthetic parts of the human body, they are used to replace human limbs, organs, or tissues to make up for the lack of human pursuit of beauty. The use of prostheses in medical cosmetology is diverse, with its advantages of plasticity and compatibility with autologous tissues having attracted many beauty professionals. Choosing a suitable prosthesis is particularly important for plastic and cosmetic effects. In the following cases, prosthetic materials have been widely used in plastic and cosmetic repair.

**Rhinoplasty.** It is to implant the prosthesis into the nose through surgery to achieve the effect of raising the back of the nose and shaping the overall shape of the nose. Silicone prosthesis is an ideal material for rhinoplasty, which has good compatibility, little irritation to nasal tissues, low price, and easy carving [38,70]. After the rhinoplasty operation, the recipient should try to minimize adverse stimuli such as touch and impact at the surgical site to ensure the normal transformation of the capsular fibers [70].

**Breast enhancement.** A high-quality silicone prosthesis is implanted under the pectoralis major muscle, and through the axillary incision or fold incision, with the purpose of breast augmentation, plastic surgery, and straightening achieved. Compared with silicone prostheses, silicone gel prostheses are soft, and insoluble in water and inorganic acids [71]. They feel realistic, soft, and blend with human anatomy. The adaptability is great and the choice is large. The prosthesis has a supporting effect, which can correct the sagging breast while plumping the breast [71].

**Plumping forehead.** The plumping forehead has been widely used in the repair of soft tissue defects and plastic surgery. It refers to cutting a small opening in the depression of the forehead and implanting the silicone prosthesis or expansion into the receiving area to improve the appearance. The advantages of using a prosthesis to plump the forehead are easy operation, safe and stable performance, good molding, and easy removal [72].

**Plumping buttock.** The use of prostheses to enlarge the buttocks is to cut a small hole in the gluteal groove under local anesthesia, and implant the prosthesis in the upper gluteus maximus and the lower gluteus maximus [73]. The muscle acts as a support for the prosthesis and prevents the prosthesis from moving downwards. In addition, because the muscles and subcutaneous fat cover the surface of the prosthesis, the feel is more natural and the shape more perfect [73,74].

**Maxillofacial reconstruction.** Ceramic and metallic implants are used to fabricate patient-specific bone grafts to promote bone regeneration. Brie et al. [75] reported a technique for making bioceramic implants to match each patient’s craniofacial bone defect. According to the CT data of patients, implants were made by a stereolithography technique using hydroxyapatite and resin. The results showed good esthetic and stability.

#### 2.2.2. Injectable Materials

**Hyaluronic acid.** Hyaluronic acid is a polysaccharide that is a natural ingredient in the skin. It exists in the collagen fibers of the dermis in the form of colloids, which acts as a water lock and makes the skin look young and full. It has been widely used to fill the wrinkles of the decree lines, corners of the mouth, forehead, and eyebrows, and can even be used to repair the nose, lips, chin, acne, and chickenpox crypt scar correction [29,76]. In the past, hyaluronic acid was derived from the connective tissues of animals, such as cockscomb, umbilical cord, eyeballs, cartilage, etc. The composition and structure of hyaluronic acid are the same as those of human hyaluronic acid and will not cause immune rejection. The disadvantage of injecting hyaluronic acid is that it cannot be maintained for a long time after being absorbed by the body [30]. In general, the effect is maintained between six and nine months, but this is related to different injection sites. Individual physiological differences, such as lip shaping, may last a shorter time than other facial areas.

**Aqumid (polyacrylamide)**. The main component of Aqumid filler is polyacrylamide gel, which has a longer cosmetic effect than hyaluronic acid preparations. Negredo et al. [77] conducted a clinical trial and injected it into the skin of the face of AIDS patients and found that it had a good cosmetic effect. It is reported that the cosmetic effect of this product can last 7–10 years. The main indications are lips, nasolabial fold, and malar area [78]. These fillers may cause facial defects such as facial swelling. Once the hidden danger appears, these fillers cannot be removed.

**Artecoll.** Artecoll is a new type of medical plastic cosmetic agent that contains two safe ingredients: PMMA microspheres and collagen. When Aibefu is injected into the area where wrinkles need to be removed, collagen immediately takes effect and the wrinkles disappear. It is well known that the formation of wrinkles in human faces is caused by the breakdown of collagen under the skin, and therefore skin collagen regeneration is an important way to eliminate facial wrinkles [79,80]. This material can stimulate the regeneration of collagen under the skin, restore the original thickness of the skin, eliminate wrinkles and other aging signs, and can continuously regenerate collagen to replace the lost collagen and maintain the dynamic balance of subcutaneous collagen.

**Autologous fat.** Due to the limited space provided by the chest, we cannot inject too many fat cells at a time, and otherwise, part of the fat cells will be absorbed by the body. This needs to be divided into several times. Requirements for the doctor’s experience and equipment are high, and surgery must be performed in a formal medical and cosmetic institution. For the problem of self-absorption, it may be necessary to perform another operation [81]. The clinical application of autologous fat is not only used for routine cosmetic treatment, such as wrinkle removal but also widely used for congenital defects, trauma, or reconstruction of surgically removed tissues, such as breast collapse after breast tumor surgery [82] and soft tissue deficiency after maxillofacial surgery [83].

**Collagen.** Collagen is the main component of connective tissue, ligament, and tendon [27,84]. At present, the main sources of collagen biomaterials are bovine collagen, pig collagen, and human collagen. Now there are a variety of bovine collagen and pig collagen products on the market, which can be used in scars, crow’s feet, and periorbital wrinkles [85]. However, it has been reported that bovine collagen has a high incidence of rejection [86]. Human collagen materials have been used in clinical practice to delay the degradation of collagen lysine residues by glutaraldehyde crosslinking, mainly for the treatment of deep scars and wrinkles [87,88]. Human collagen materials have shown a good clinical effect, but its long-term efficacy remains to be confirmed by clinical research.

**Fibrin glue.** Fibrin glue has a long and diverse record in clinical application. In general, the product has been proven to be safe, except for very few allergic reactions and very few allergic reactions [89]. Fibrin glue is effective in seroma prevention [90], face-lift surgery [91], and skin grafts [92]. However, the efficacy of fibrin glue lacks adequate powered prospective trails. In the future, prospective, randomized, controlled clinical trials should be conducted to determine the efficacy of fibrin glue in routine plastic surgery.

## 3. Development and Application of 3D Manufacturing Technology in Biomaterials for Plastic Surgery

The three-dimensional manufacturing technology, started in the 1980s, is a new application technology combining computer 3D digital imaging technology and multi-layer continuous printing technology. Through 3D scanning and computer-aided design, a 3D digital model was designed. Then, models can be input into the printer in the STL format file and fabricating complex and precise structures by stacking biomaterials layer-by-layer [13]. The 3D manufacturing has a strong potential in regenerative medicine to fabricate customized scaffolds for both hard tissue and soft tissue regeneration [93,94,95,96,97].

At present, the methods of 3D manufacturing technology can be divided into traditional construction and additive manufacturing forming technology. Traditional construction includes the solvent casting method, particle leaching method, freeze-drying method, and freeze-gel methods, which are simple and economical [98]. Additive manufacturing forming technology includes fused deposition modeling (FDM) and direct 3D bio-fabrication [98]. The advantage of additive manufacturing forming technology is that the scaffold can be customed for patients according to their characteristics, and scaffold materials, seed cells, and growth factors can be prepared accurately [99]. Traditionally, FDM technology based on extrusion molding can be used to fabricate three-dimensional porous scaffolds layer by layer. The main disadvantage is that cells must be inoculated to the scaffolds after printing. Chhaya et al. [100] added human umbilical vein endothelial cells (HUVEC) into PLLA scaffolds and implanted them subcutaneously into nude mice without a thymus. Angiogenesis and adipose tissue formation were observed in all breast stents. The 3D bioprinting, based on tissue engineering and stem cell research, using living cells and growth factors as printing materials, can further realize the formation of bioactive tissues or organs in vitro [13,14]. Current research on bio-fabrication focuses on how to make cells stay alive and differentiate into the tissue after printing [101].

The requirements and limitations for 3D manufacturing materials such as biocompatibility, biodegradability, pore connectivity, pore size, porosity, and mechanical properties should be considered in the fabrication of appropriate 3D scaffolds [102]. Biocompatibility and biodegradability are the most important characteristics of scaffolds, which ensure that they are degraded into non-toxic products while leaving only ideal living tissues. In addition, the pore connectivity, pore size, and porosity of the scaffold should be appropriate for cellular attachment, proliferation, and differentiation. Finally, the mechanical stability of the scaffold must be well constructed to withstand daily activities and normal physical movement [102,103]. The materials used for 3D manufacturing scaffolds can be divided into naturally derived materials and synthetic polymers. Naturally derived materials mainly include alginate, chitosan, collagen, fibronectin, and hyaluronic acid. Compared with synthetic polymers, naturally derived materials can simulate genuine ECM, enhance cell adhesion, and regulate cell proliferation more effectively [104]. As for synthetic polymers, the most often used ones are poly(e-caprolactone) (PCL) and poly (D, L-lactic-co-glycolic acid) (PLGA). Although natural materials are beneficial to cellular processes, synthetic polymers have higher mechanical strength, higher processability, and controllable degradation rate [104,105].

**Figure 2:**
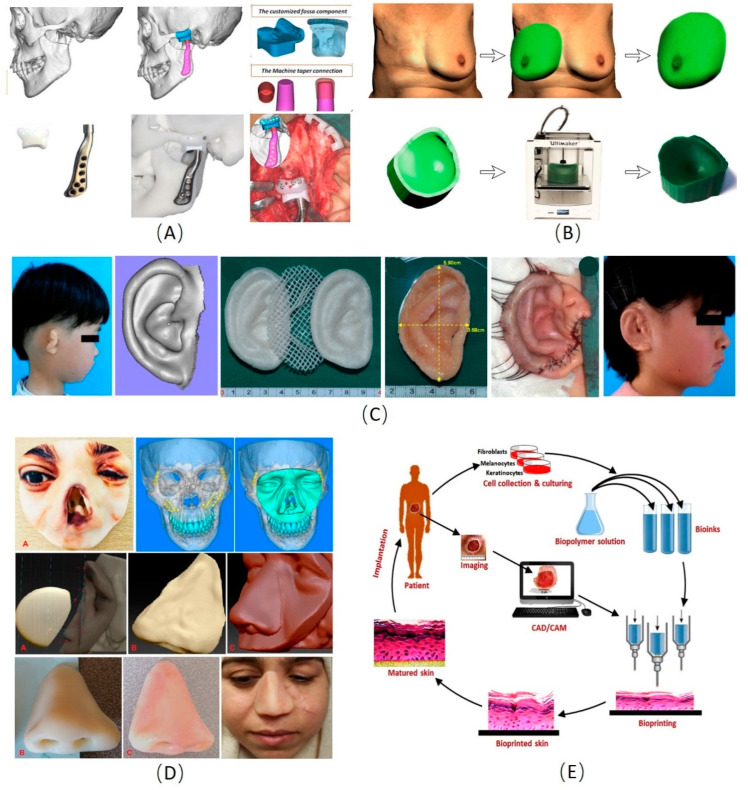
Examples of the applications of three-dimensional (3D) manufacturing technology in plastic and cosmetic surgery [108,109,110,111,112,113,114,115,116,117,118,119,120,121,122,123,124,125,126]. (**A**) The 3D manufacturing technology for temporomandibular joint reconstruction [111]. (**B**) Design of a patient-specific template for unilateral breast reconstruction. The contralateral breast is virtually isolated and mirrored based on 3D photography. Extrusion of the outer rim of the virtual breast provides a flat base. Finally, the design is printed using a 3D printer [125]. (**C**) The process of 3D printing PCL and PGA scaffold and autologous residual ear cartilage co-culture to generate an individualized auricular implant [58]. (**D**) The process of obtaining a nasal defect model and make prosthesis to repair the nasal defect by the 3D manufacturing technology [113]. (**E**) Steps in the fabrication of bioprinted skin. Various cells would be collected from the patient and grow and multiply in the cell culture system. A suitable biopolymer is mixed with the cells and the formed bioink is fed to the bioprinting system. Features of the wound are captured and a 3D structure is reconstructed using CAD/CAM approaches. According to the 3D pattern, wound tissue will be reconstructed, allowed for maturation in vitro and implanted back to the patient [121]. PLA: Polylactic acid; PGA: Polyglycolic acid; CAD/CAM: Computer-aided design and computer-aided manufacturing.

Currently, the 3D manufacturing technology has a wide range of applications in the plastic cosmetic area, such as craniofacial and maxillofacial bone reconstruction [98,106,107], ear and nose reconstruction, skin printing, breast reconstruction, and other fields, especially in the production of bone substitutes, personalized prostheses, and prostheses (Figure 2) [14,108,109,110].

### 3.1. Cranio-Maxillofacial Reconstruction

Tumors, trauma, inflammation, congenital malformations, and other factors can destroy the continuity and integrity of the craniofacial bone, resulting in abnormalities or dysfunction of the craniofacial surface. The traditional repair methods mainly include bone grafting and bone replacement implantation, but the maxillofacial anatomy is complex and individual differences are obvious. Standardized bone substitutes are not well suited for every patient. Implants with limited functions and short service life can easily cause problems [108]. Autologous bone transplantation takes a long time, prone to infection, pain, and other complications, with postoperative appearance recovery being poor. Accordingly, the advent of three-dimensional printing technology makes up for the deficiencies of traditional surgical methods. At present, the application of 3D manufacturing technology in craniofacial and maxillofacial bone repair and reconstruction has gradually matured [108,109,110,111].

### 3.2. Ear and Nose Reconstruction

Otolaryngeal defects or deformities caused by trauma, tumors, or congenital dysplasia will have a great impact on the appearance of the patient and seriously affect the quality of life of the patient. In the past, soft tissue expanders combined with autologous cartilage or prostheses were used for repair and reconstruction, but the postoperative appearance was not ideal, and complications such as infection and rejection were prone to occur. At present, the technology of using 3D manufacturing technology to customize personalized ear-nose prosthesis is relatively mature [110,112,113]. After collecting data with photogrammetric equipment, a portable desktop 3D printer can produce high-quality silicone soft prostheses in a short time [114,115]. Compared with traditional prostheses, 3D printed ear and nose repair prostheses have huge advantages in terms of material design flexibility and personalized size customization [115,116].

### 3.3. Skin 3D Manufacturing

Due to the limited sources and applications of autologous/allogeneic skin, scientists have been looking for ideal skin substitutes. Now due to the lack of epidermal or dermal component support, more and more tissue-engineered skin is being used. However, after tissue engineering skin transplantation, complications such as infection and scar contracture are prone to occur, and skin reconstruction cannot be achieved. The 3D printed skin provides a new direction for the research on skin substitutes [117]. Using a three-dimensional scanner to measure and record the position of skin tissue layers and specific cells, it is expected to customize synthetic artificial skin to meet the biological function design of the skin [118,119,120,121].

### 3.4. Breast Shaping and Reconstruction

Before breast reconstruction, it is necessary to accurately measure the various aesthetic indicators of the double breasts, such as position, volume, breast height, and position of the nipple and areola complex. CT or MRI three-dimensional imaging results are more accurate and can be used for the estimation and adjustment of double breast symmetry [122,123]. Breast reconstruction can be divided into several ways: Autologous tissue transplantation reconstruction, prosthesis implantation reconstruction and autologous tissue, and prosthesis combined reconstruction. Using three-dimensional printing technology, density and layering can be used to accurately control the volume and shape of the prosthesis [123]. The density stratification makes the base more stable, and also ensures that the content of the prosthesis matches the distribution of the chest wall ligaments, thereby providing upward tension for the prosthesis and ensuring that the breast is slightly cambered and upward [124,125]. Defazio et al. [126] produce breast and nipple-areola prostheses using a 3D printer. These prostheses have been successfully used for breast reconstruction after breast cancer surgery, greatly reducing the incidence of postoperative complications. At the same time, the postoperative appearance is natural and beautiful, and patient satisfaction is high.

## 4. Conclusions and Perspective

Although biomedical materials have good biocompatibility, certain biological activity, and no toxic and side effects, they all have certain limitations. With the wide application of these biomedical materials, research on biomedical materials is getting deeper and deeper, with these shortcomings having gradually overcome. The 3D printing technology has been widely used in plastic surgery, especially craniofacial reconstruction, ear and nose reconstruction, skin printing, and breast reconstruction. Combined with research results such as regenerative medicine, stem cells, and tissue engineering, “biological 3D manufacturing” is carried out under certain conditions, which has the appearance and structure of human tissues and organs, and at the same time gives some physiological functions. In the end, it is expected to solve the limitations of autotransplantation or allotransplantation. The 3D manufacturing technology also provides good tolerance, reproducibility, and a patient-specific model for surgical training. The emerging research of 3D biomaterial printing has led to the development of biocompatible scaffolds, which have the potential advantage for tissue regeneration. The main limitations of using 3D manufacturing technology include time and cost, which may be offset by the reduced operating time and interdepartmental collaboration to spread internal printing costs.

The current literature shows promising results of biomaterials and 3D manufacturing in plastic surgery, but it has not been confirmed by large-scale studies or randomized controlled trials. Ultimately, further research and progress in biomaterials and 3D manufacturing technology should be supported, as it has the potential for surgical satisfaction in plastic surgery. The future development trend of biomedical materials should include two aspects. The first aspect is the development of composite materials, which can overcome the shortcomings of a single material and improve the overall performance of the material. The second aspect is the development of new regenerative biomaterials, such biomaterials that can induce the regeneration of damaged tissues or organs. These materials will promote the repair of damaged tissues or organs to achieve permanent repair of damaged tissues.

## Figures and Tables

**Table 1:** Overview of natural biomaterials related to plastic surgery.

Name	Source	Application Mode	Application Site	Advantages	Disadvantages
**Natural Biomaterials**					
Bio-protein glue [15,16,17,18]	Blood from animals	Injectable liquid	Hemostasis of venule hemorrhage in burn patients	Good biocompatibility	The application range is narrow
Decellularized tissue [19,20,21,22,23,24,25,26]	Dermis, valve, and other tissue from human or animals	Biological patch	Skin transplantation in burn patients	Good biocompatibility	High cost
Collagen [27,28]	Connective tissue of animals, such as bovine achilles tendon	Injectable liquid	Facial soft tissue filler for crow’s feet, periorbital wrinkles, and deep scars	Good biocompatibility; can interact with cells; good mechanical properties; moisture retention; low immunogenicity; biodegradability	Rejection reaction; possible disease transfection
HA [29,30,31,32,33,34,35,36]	cockscomb, umbilical cord, eyeballs, and cartilage	Injectable liquid	Fillers for wrinkles of the decree lines, corners of the mouth, forehead, eyebrows, nose, lips, chin, acne, and chickenpox crypt scar	Minimal rejection reaction	Mild swelling; rapid degradation

HA: Hyaluronic acid.

**Table 2:** Overview of synthetic polymers related to plastic surgery.

Name	Application Mode	Application Site	Advantages	Disadvantages	Molecular Structure/Formula
**Organic Polymer Materials**					
Silicone [37,38,39,40,41]	Prosthesis	Rhinoplasty, breast enhancement, plumping forehead and lumping buttock	Heat resistance, cold resistance, non-toxicity, biological aging resistance, physiological inertia, little response to human tissues, and good physical and mechanical properties	Strong hydrophobicity, poor antibacterial properties, and aging problems	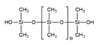
ePTFE [42,43,44,45]	Prosthesis	Ear reconstruction, rhinoplasty	Good biocompatibility, good tensile strength	Non-biodegradable, poor antibacterial properties	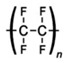
PMMA [46,47,48,49,50,51]	Injectable liquid	Facial wrinkles, fill in acne scars, correct nipple depressions, highlight chins, and swell cheeks	long-lasting	Bead protrusions, allergic reactions, telangiectasia, and granuloma formation	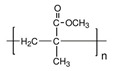
High-density polyethylene [52,53,54,55,56]	Prosthesis	Repair of cheeks, orbital arch, orbital floor, upper and lower jaws, cheekbones, temporal, and ears	Good biocompatibility, rough, convergent, and porous structure	High risk of infection, poor mechanical properties	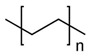
PLA and PLGA [57,58,59,60]	Prosthesis; sutures	Surgical absorbable sutures, bone fixation plates, screws	Good biocompatibility, biodegradability	Rapid biodegradation	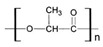 & 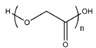
**Ceramic Materials**					
Hydroxyapatite [61,62,63,64]	Prosthesis	Substitute for large area bone defects such as oral and maxillofacial regions, substitutes for teeth	Good biocompatibility, biodegradability, low toxicity	High brittleness, low strength, and poor toughness	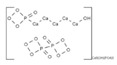
Bioglass materials [65,66]	Prosthesis	Bone repair and regeneration	Good biocompatibility and bone binding ability	Low mechanical strength, high brittleness, poor bending strength, and compressive strength	E.g. 45S5 Bioglass^®^ (developed by L.L. Hench): 45 wt% SiO_2_, 24.5 wt% CaO, 24.5 wt% Na_2_O, and 6.0 wt% P_2_O_5_.

ePTFE: Expanded polytetrafluoroethylene; PMMA: Polymethyl methacrylate; PLA: Polylactic acid; PGA: Polyglycolic acid.
